# Establishment of reference standards for multifaceted mosaic variant analysis

**DOI:** 10.1038/s41597-022-01133-8

**Published:** 2022-02-03

**Authors:** Yoo-Jin Ha, Myung Joon Oh, Junhan Kim, Jisoo Kim, Seungseok Kang, John D. Minna, Hyun Seok Kim, Sangwoo Kim

**Affiliations:** 1 Department of Biomedical Systems Informatics, Brain Korea 21 Project, Yonsei University College of Medicine, Seoul, 03722 Republic of Korea; 2 Severance Biomedical Science Institute, Brain Korea 21 Project, Yonsei University College of Medicine, Seoul, 03722 Republic of Korea; 3 Hamon Center for Therapeutic Oncology Research, University of Texas Southwestern Medical Center, Dallas, Texas USA

**Keywords:** Standards, Genomics, Next-generation sequencing

## Abstract

Detection of somatic mosaicism in non-proliferative cells is a new challenge in genome research, however, the accuracy of current detection strategies remains uncertain due to the lack of a ground truth. Herein, we sought to present a set of ultra-deep sequenced WES data based on reference standards generated by cell line mixtures, providing a total of 386,613 mosaic single-nucleotide variants (SNVs) and insertion-deletion mutations (INDELs) with variant allele frequencies (VAFs) ranging from 0.5% to 56%, as well as 35,113,417 non-variant and 19,936 germline variant sites as a negative control. The whole reference standard set mimics the cumulative aspect of mosaic variant acquisition such as in the early developmental stage owing to the progressive mixing of cell lines with established genotypes, ultimately unveiling 741 possible inter-sample relationships with respect to variant sharing and asymmetry in VAFs. We expect that our reference data will be essential for optimizing the current use of mosaic variant detection strategies and for developing algorithms to enable future improvements.

## Abstract (summary)

**Table:** 

Measurement(s)	genotype
Technology Type(s)	DNA sequencing
Factor Type(s)	genotyping
Sample Characteristic - Organism	Homo sapiens
Sample Characteristic - Environment	cell line

Machine-accessible metadata file describing the reported data: 10.6084/m9.figshare.16970041

## Background & Summary

After conception, postzygotic mutations continuously occur throughout life in humans, causing somatic mosaicism in an individual^1,2^. The variant type, time of origination, and locations of the mosaic mutations result in unique mosaic patterns in a combinatorial manner and further affect phenotypes, including various noncancerous diseases^3–12^. Several efforts have, thus, been made to identify the mutational landscape and mechanisms underlying the mosaic mutations^13–17^.

From a technical aspect, the accurate detection of mutations is at the core of the mosaicism research. To date, conventional bulk sequencing has mainly been exploited by utilizing or modifying variant detection algorithms developed for calling clonal variants, such as cancer mutations^6,18,19^. However, successful application to mosaicism has been obstructed by many challenges, such as low variant allele frequencies (VAF < 10%)^14,17,20,21^ and ambiguity in the use of a control (e.g., variants can exist in control samples by shared lineages in development)^14,17^. Moreover, fundamentally, there is a severe lack of platforms or materials, known as reference standards, that can be used to measure the detection accuracy of given algorithms^22^, thereby amplifying the confusion regarding the optimal use of tools or algorithms and their reliability. Constructing a standard reference is, thus, a critical first step and serves as the basis for analytical validation and benchmarks for germline and somatic mutations^23–30^. Furthermore, securing a reference standard for mosaic mutations is urgently needed to enable more advanced research.

**Fig. 1:**
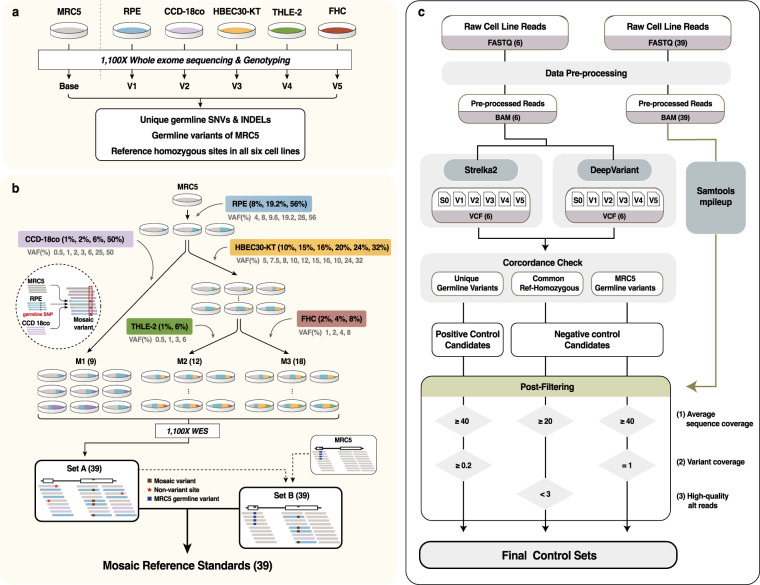
Overall workflow of mosaic reference standard construction. (**a**) Schematic of the genotyping of six cell lines used as materials. (**b**) Construction of 39 mosaic reference standards by mixing genetic materials of the six cell lines. Thirty-nine pairs of Set A and Set B were generated by different combinations and proportions of the six cell lines. Set A, sequencing data of the original mixtures; Set B, MRC5 sequencing data with replacement of sequences of variant sites from Set A. (**c**) Pipeline to generate positive and negative controls in the reference standards. After BAM file preprocessing, candidates for controls were cross-checked using Strelka2 and DeepVariant. Final control sets were fixed with three post-filters using raw read counts (pileup) of 39 mixtures and MRC5 WES data. WES Whole exome sequencing.

Herein, we generated robust, large-scale, and cell line mixture-based reference standards using 386,613 single-nucleotide variants (SNVs) and insertion-deletion mutations (INDELs) as positive controls and 35,133,353 negative control positions. The workflow for generating the standard materials and for variant site identification is displayed in Fig. 1. The overall idea for the construction aligns with our previous study^31^, as unique germline variants among independent genotypes serve as mosaic variants when mixed in the desired proportions. Initially, six normal cell lines (MRC5, RPE, CCD-18co, HBEC30-KT, THLE-2, and FHC) were prepared and sequenced (1,100 × WES) to identify a set of mutually exclusive germline variants. We confirmed those germline variants to be unique in only one cell line with explicit reference homozygous genotypes in the other five (see Methods). When MRC5 was employed as an internal reference, each of the five remaining cell lines (RPE, CCD-18co, HBEC30-KT, THLE-2, and FHC) had a unique set of variants among all, and were called V1 to V5, respectively (Fig. 1a; see Table 1 for the full list). When mixed with MRC5 in different proportions, these unique variants are presented as mosaic mutations at designated VAFs.

**Table 1:** Variant set of five cell lines.

Variant type	Zygosity	Variant Set					Total
		V1	V2	V3	V4	V5	
**SNV**	**Het**	2,698	6,158	2,058	2,127	5,825	18,866
	**Hom**	133	350	188	129	349	1,149
**INDEL**	**Het**	89	212	65	60	173	599
	**Hom**	5	12	7	7	16	47
**Total**		2,925	6,732	2,318	2,323	6,363	20,661

RPE, CCD-18co, HBEC30-KT, THLE-2, and FHC, represents V1-V5, respectively. Het heterozygous, Hom homozygous.

The mixing procedure was systematically designed to cover a wide range of VAFs and various variant sharing scenarios (Fig. 1b). Importantly, common (i.e., acquired before the lineage separation of two samples) and lineage-specific (i.e., acquired after the lineage separation) variants compose an internal hierarchical structure of mosaic genotypes in an organism, mimicking the cumulative aspect of mosaic variant acquisition from early (e.g., developmental stage) to late (e.g., recent). RPE was mixed into the internal reference (MRC5) at three different ratios (8, 19.2, and 56%) to enable the presentation of the variants in RPE (V1) at six different VAFs (4, 8, 9.6, 19.2, 28, and 56%), depending on the zygosity (hetero- or homozygous). Similarly, CCD18-co (V2) and HBEC30-KT (V3) were added into the MRC5/RPE mixture at four and six different ratios, respectively. Finally, THLE-2 (V4) and FHC (V5) were added into the MRC5/RPE/HBEC30 mixture at two and three different ratios, respectively (Fig. 1b upper). After the procedure, three final classes of products were generated: M1 (the mixture MRC5/RPE/CCD18-co), M2 (MRC5/RPE/HBEC30-KT/THLE-2), and M3 (MRC5/RPE/HBEC30-KT/FHC). M1 contains the variant sets V1 and V2; M2 contains V1, V3, and V4; and M3 contains V1, V3, and V5, whose VAFs varied according to the mixing ratios within the classes. Of the 12 (3 in RPE × 4 in CCD-18co), 36 (3 in RPE × 6 in HBEC30 × 2 in THLE-2), and 54 (3 in RPE × 6 in HBEC30 × 3 in FHC) possible products in classes M1–M3, 9, 12, and 18 were selected for redundancy and covering efficiency, and subsequently sequenced to ultra-high coverage (1,100×) whole-exome sequencing (WES; see Table 2 for the full list). Overall, 9,657, 7,566, and 11,606 positive control variants were included in M1–M3, respectively, with a wide range of VAFs (0.5–56%), particularly focusing on low frequencies (<10%) (Table 2).

**Table 2:** The compositions and VAFs of variant sets of thirty-nine products.

Category	Product	VAF of variant set (Het%/Hom%)					# SNV	# INDEL	Total
		V1	V2	V3	V4	V5			
**M1**	**M1-1**	4.0/8.0	1.0/2.0	—	—	—	9,339	318	9,657
	**M1-2**	4.0/8.0	3.0/6.0	—	—	—	9,339	318	9,657
	**M1-3**	4.0/8.0	25.0/50.0	—	—	—	9,339	318	9,657
	**M1-4**	9.6/19.2	1.0/2.0	—	—	—	9,339	318	9,657
	**M1-5**	9.6/19.2	3.0/6.0	—	—	—	9,339	318	9,657
	**M1-6**	9.6/19.2	25.0/50.0	—	—	—	9,339	318	9,657
	**M1-7**	28.0/56.0	1.0/2.0	—	—	—	9,339	318	9,657
	**M1-8**	28.0/56.0	3.0/6.0	—	—	—	9,339	318	9,657
	**M1-9**	4.0/8.0	0.5/1.0	—	—	—	9,339	318	9,657
**M2**	**M2-1**	4.0/8.0	—	5.0/10.0	0.5/1.0	—	7,333	233	7,566
	**M2-2**	4.0/8.0	—	5.0/10.0	3.0/6.0	—	7,333	233	7,566
	**M2-3**	4.0/8.0	—	8.0/16.0	0.5/1.0	—	7,333	233	7,566
	**M2-4**	4.0/8.0	—	8.0/16.0	3.0/6.0	—	7,333	233	7,566
	**M2-5**	9.6/19.2	—	5.0/10.0	0.5/1.0	—	7,333	233	7,566
	**M2-6**	9.6/19.2	—	5.0/10.0	3.0/6.0	—	7,333	233	7,566
	**M2-7**	9.6/19.2	—	8.0/16.0	0.5/1.0	—	7,333	233	7,566
	**M2-8**	9.6/19.2	—	8.0/16.0	3.0/6.0	—	7,333	233	7,566
	**M2-9**	28.0/56.0	—	5.0/10.0	0.5/1.0	—	7,333	233	7,566
	**M2-10**	28.0/56.0	—	5.0/10.0	3.0/6.0	—	7,333	233	7,566
	**M2-11**	28.0/56.0	—	8.0/16.0	0.5/1.0	—	7,333	233	7,566
	**M2-12**	28.0/56.0	—	8.0/16.0	3.0/6.0	—	7,333	233	7,566
**M3**	**M3-1**	4.0/8.0	—	7.5/15.0	—	1.0/2.0	11,251	355	11,606
	**M3-2**	4.0/8.0	—	7.5/15.0	—	2.0/4.0	11,251	355	11,606
	**M3-3**	4.0/8.0	—	7.5/15.0	—	4.0/8.0	11,251	355	11,606
	**M3-4**	4.0/8.0	—	12.0/24.0	—	1.0/2.0	11,251	355	11,606
	**M3-5**	4.0/8.0	—	12.0/24.0	—	2.0/4.0	11,251	355	11,606
	**M3-6**	4.0/8.0	—	12.0/24.0	—	4.0/8.0	11,251	355	11,606
	**M3-7**	9.6/19.2	—	7.5/15.0	—	1.0/2.0	11,251	355	11,606
	**M3-8**	9.6/19.2	—	7.5/15.0	—	2.0/4.0	11,251	355	11,606
	**M3-9**	9.6/19.2	—	7.5/15.0	—	4.0/8.0	11,251	355	11,606
	**M3-10**	9.6/19.2	—	16.0/32.0	—	1.0/2.0	11,251	355	11,606
	**M3-11**	9.6/19.2	—	16.0/32.0	—	2.0/4.0	11,251	355	11,606
	**M3-12**	9.6/19.2	—	16.0/32.0	—	4.0/8.0	11,251	355	11,606
	**M3-13**	28.0/56.0	—	10.0/20.0	—	1.0/2.0	11,251	355	11,606
	**M3-14**	28.0/56.0	—	10.0/20.0	—	2.0/4.0	11,251	355	11,606
	**M3-15**	28.0/56.0	—	10.0/20.0	—	4.0/8.0	11,251	355	11,606
	**M3-16**	28.0/56.0	—	16.0/32.0	—	1.0/2.0	11,251	355	11,606
	**M3-17**	28.0/56.0	—	16.0/32.0	—	2.0/4.0	11,251	355	11,606
	**M3-18**	28.0/56.0	—	16.0/32.0	—	4.0/8.0	11,251	355	11,606
**Total**							**374,565**	**12,048**	**386,613**

M1, M2, and M3 refer to the three classes depending on the constituent cell lines and 9, 12, 18 products were generated respectively, according to different mixing ratio. V1 RPE, V2 CCD-18co, V3 HBEC30-KT, V4 THLE-2, V5 FHC, VAF variant allele frequency, Het heterozygous, Hom homozygous.

Two different types of reference standards are required to enable complete measurement of mosaic detection accuracy, which differ based on the definition of negative controls. Unlike conventional somatic mutations, calling of mosaic variants is susceptible to two different types of errors: (1) calling non-variant sites (e.g., reference allele) and (2) calling germline variants, the latter of which is caused by the unreliability of controls (e.g., variants shared in control samples). Therefore, we provide two different versions of the final sets—set A and set B (Fig. 1b lower). Set A is the sequencing data of the original materials, M1–M3, which uses 35,113,417 non-variant sites as negative controls. Set B is processed data, where the sequencing data (BAM) of non-variant sites are replaced by those of the internal reference (MRC5) to contain 19,936 germline variants; this is because the original germline compositions of MRC5 are altered in set A by the mixing procedure. Accordingly, testing should be carried out in both sets. The final list of negative controls is presented in Table 3.

**Table 3:** Count of negative controls in final sets.

Version of final sets	Negative control type	Variant type	Zygosity	Count
**Set A**	Non-variant sites	—	—	35,113,417
**Set B**	Germline variants	SNV	Het	11,734
			Hom	7,763
		INDEL	Het	296
			Hom	143

Different types of negative controls are included in the two version of the final sets, Set A and Set B. Het heterozygous, Hom homozygous.

Finally, our reference standards allow testing under various realistic biological scenarios by mimicking the structure of multiple lineages in the accumulation of mosaic mutations. There are 741 possible ways to select two within thirty-nine reference data (9 M1, 12 M2, 18 M3), each of which provides distinct inter-sample relationships of variant sharing and their VAF distributions, providing a truth sets for shared and nonshared mosaic variant detection. For example, M1 and M2 share the variant set V1 in varied VAF pairs in respect to the selection of the data, whereas V2 is unique in M1, and V3 and V4 are unique in M2. Likewise, M2 and M3 share V1 and V3. In this regard, M2 and M3 are considered closer in the lineage as they have a more recent common ancestor, which can be exploited in more advanced algorithms. The target VAFs display the tendency to decrease in later mutations^1,32,33^. Exceptions caused by the asymmetric doubling of cells and active replication of stem cells or progenitor cells are also considered^3,16^. Owing to these features, our data constitute one of the most comprehensive, versatile, and robust reference standards ever constructed for variant analysis.

## Methods

### Sample collection and preparation

Six immortalized normal cell lines (MRC5, RPE, CCD-18co, HBEC30-KT, THLE-2, FHC) were chosen for the construction the reference standards, after confirming their stable genotypes with neutral ploidy, (see Technical Validation). FHC and THLE-2 cells were purchased from the American Type Culture Collection (ATCC). RPE was purchased from Lonza Bioscience. MRC5 and CCD-18co were purchased from the Korea Cell Line Bank. HBEC30-KT is a transformed cell line of HBEC with two genetic alterations (CDK4, hTERT)^34^, and its genomic DNA is available under request. The absence of mycoplasma contamination in all cell lines was verified using the e-Myco VALiD Mycoplasma PCR Detection Kit (LiliF Diagnostics). Cell line authentication was performed using the PowerPlex 18D System (Promega, Cosmogenetech Co., Ltd.) to detect 17 short tandem repeat (STR) loci. The resulting STR profiles were cross-compared and matched with deposited STR information. Since STR profile for RPE, which we purchased from Lonza, was not provided, we attached its STR analysis results along with other cell lines in Online-only Table 1.

All cell lines were cultured in a humidified environment in the presence of 5% CO_2_ at 37 °C. FHC cells were grown in DMEM:F12 (Gibco) with 25 mM HEPES (Gibco), 0.005 mg/mL insulin, 0.005 mg/mL transferrin, 100 ng/mL hydrocortisone, 20 ng/mL human recombinant EGF (Thermo Fisher), 10 ng/mL cholera toxin, 10% fetal bovine serum (Gibco), and 1% penicillin–streptomycin (Invitrogen). THLE-2 cells were grown in BEBM (Lonza) supplemented with BEGM Bronchial Epithelial SingleQuots Kit (excluding GA-1000, Lonza), 10% fetal bovine serum, and 1% penicillin–streptomycin. RPE cells were grown in RtEBM (Lonza) supplemented with RtEGM SingleQuots Supplement Pack (Lonza) and 1% penicillin–streptomycin. MRC5 cells were grown in MEM (Gibco) with 25 mM HEPES, 25 mM NaHCO_3_, 10% fetal bovine serum, and 1% penicillin–streptomycin. CCD-18co cells were grown in DMEM with L-glutamine (300 mg/L, Gibco), 25 mM HEPES, 25 mM NaHCO_3_, 10% fetal bovine serum, and 1% penicillin–streptomycin. HBEC30-KT cells were grown in ACL4 media comprising RPMI 1640 medium supplemented with 0.02 mg/mL insulin, 0.01 mg/mL transferrin, 25 nM sodium selenite, 50 nM hydrocortisone, 10 mM HEPES, 1 ng/mL EGF, 0.01 mM ethanolamine, 0.01 mM O-phosphorylethanolamine, 0.1 nM triiodothyronine, 2 mg/mL BSA, 0.5 mM sodium pyruvate, 2% fetal bovine serum, and 1% penicillin–streptomycin.

To achieve the target ratios, mixing was carried out at a DNA level based on the pre-calculated quantities (see Table 2 for final mixture ratios). Genomic DNA was extracted using a QIAamp DNA Mini Kit, according to the manufacturer’s instructions (QIAGEN). A total of 39 mixtures were generated by mixing the genomic DNAs from the six cell lines (see **Summary** for the procedure). After mixing the genomic DNAs according to the pre-calculated quantities on ice, the mixtures were briefly vortexed, centrifuged, and stored at −20 °C.

### Whole exome sequencing

Exome capture was carried out for six cell lines and 39 mixtures using SureSelect Human All Exon V6 (Agilent Technologies, Inc., CA, USA). To minimize duplicate reads in ultra-deep sequencing, sequencing libraries were constructed two (cell lines) to four (mixture) times for each sample. The quantities of the constructed libraries were evaluated using the 2100 Bioanalyzer Systems (Agilent Technologies, Inc). WES was conducted for the six initial cell lines and 39 mixtures using Illumina NovaSeq. 6000 (Theragen Bio Inc.), with targeted read depth of 1,100×.

### Processing of the sequencing data

WES reads in FASTQ data were merged and preprocessed using fastp^35^ (0.20.0) to trim overrepresented sequences, such as poly G and adaptors. Reads with low complexity (<30%) were filtered out. The overall sequencing quality was inspected using FastQC (version 0.11.7). All passed reads were aligned to the GRCh38 reference genome using BWA-MEM^36^ (0.7.17). Post-processing, including read group addition, marking PCR duplicates, fixation of mate information, and recalibration of base quality score was applied according to the recommendations of GATK best practices using PICARD (2.23.1) and GATK (4.1.8). We also realigned and left-aligned INDELs with GATK (3.8.1 and 4.1.5, respectively) to synchronize INDEL expression in genotyping. Qualimap 2^37^ (2.2.1) was used to calculate the sequencing coverage. The overall sequencing quality information of six cell lines and thirty-nine mixtures (set A) is shown in the Online-only Table 2, including the average sequencing coverage, mapping quality, GC contents, and filtering results during the quality control.

### Genotyping of cell lines

Genotyping of the six cell lines was carried out using two robust germline variant callers: Strelka2^38^ (2.9.10) and DeepVariant^39^ (1.0.0), as they showed high accuracy (e.g., F1 scores) for detecting germline SNVs and INDELs^26,40^, for autosomal chromosomes, except chr5 (excluded by the copy number variation (CNV) identified in HBEC30, see Technical Validation). Mutually exclusive SNVs and INDELs (i.e., variants exist in only one cell line out of six) were marked as variant sets (V1–V5, see Summary) and were further considered as mosaic variants after mixing.

For SNVs, mutually exclusive variants were collected using the following criteria: (1) variants that were called in both callers and passed the default filtration; (2) variants that were called in only one of the cell lines, with the other five cells being genotyped reference homozygous (i.e., no-call is not allowed); and (3) variants with no signs of copy number alteration (log2 copy number ratio < |0.3| from cnvkit^41^). For INDELs, similar criteria were applied with an additional rescuing procedure, where single calls (out of two callers) were manually inspected using the Integrative Genomics Viewer^42^ (IGV) for the low concordance among callers^26^. Finally, mutually exclusive variants that passed all criteria in RPE, CCD-18co, HBEC30-KT, THLE-2, and FHC were called V1, V2, V3, V4, and V5, respectively (see Summary). At the same time, positions confirmed as reference homozygous (rather than no-call) by both germline callers in all six cell lines have been collected as candidates for negative control. Also, genotyping of the internal reference (MRC5) was conducted and listed for further processes.

### Finalizing reference standard sets

Genotypes of the 39 mixtures (within M1, M2, and M3) were theoretically pre-fixed by the genotypes of the six cell lines and their mixture compositions. To finalize the reference standard sets, we conducted a series of post-filtration procedures to remove sites that significantly deviated from the expected coverage and VAFs, particularly from extrinsic and systematic errors. The procedures were applied to two difference sets: set A and set B (see Summary) (Fig. 1c).

#### Reference standard with non-variant sites as the negative control (set A)

Set A is basically the sequencing data of the 39 mixtures themselves with reference homozygous sites as negative controls that are identified from the genotyping of the six cell lines. Therefore, the finalization of set A only required a few additional filtration steps.

Preprocessed sequencing data were used for the final confirmation of control positive sites based on two filtration criteria: (1) sequencing coverage and (2) variant coverage. Regarding sequencing coverage, raw allele counts were calculated in all targeted positions using SAMtools^43^ mplileup (1.10), ignoring soft or hard clips. For each variant site, the mean coverage of the 39 samples was calculated, and low coverage sites (<40×) were removed; these sites should theoretically be variant positions but cannot be used as positive controls because of the low-sequencing coverage. The threshold (40×) for sequencing coverage was determined to secure the number of positive controls as well as the quality of the reference data. With one alternative allele in 40× position, the smallest VAF that can be generated would be 2.5%, and for all variant sets (V1-V5), the proportion of designated VAFs larger than 2.5% among the total in each variant set exceed 50% (V1: 100%, V2: 55%, V3: 100%, V4: 50%, V5: 50%). Regarding variant coverage, for each variant *v*, variant coverage was defined as (number of samples that actually harbored *v*)/(number of samples designed to harbor *v*). Variants with low variant coverage (<20%) were considered to be affected by low-sequencing efficiency and were, thus, removed. For non-variant (negative control) sites, positions with an average coverage of <20× were removed. Moreover, non-variant positions with more than three high-quality (BQ ≥ 30) alternative alleles were filtered out to prevent any interference from experimental or systematic bias (e.g., small subclones generated in the original cell lines), rather than sequencing artifacts. Consequently, sequencing artifacts are projected in VAFs under 10% in negative controls non-variant negative controls, where accurate detection of mosaic variants is hampered^20^.

#### Reference standard with germline variants as the negative controls (set B)

Unlike set A, set B requires an additional process to replace germline variant sites of mixtures with those of internal reference (MRC5). First, we generated thirty-nine baseline-bam files for set B, by down sampling the MRC5 bam file into 1,100×, with random seed for 39 times using PICARD DownsampleSam (2.23.1). Then, all reads embedding the positive control positions in each of thirty-nine of set A (e.g., V1 and V2 positions for M1 data), were extracted using bedtools^44^ (2.28.0). At the same time, MRC5 reads in the same positions were removed from the down-sampled baseline data. Finally, we merged the extracted reads from each of the thirty-nine set A with the down-sampled MRC5 data where the reads in the exactly same regions were removed. Before the replacement, we verified that the sequenced fragment length, GC content, and quality of bases were comparable for the two types of data, WES reads of MRC5, and 39 mixtures. Consequently, mosaic variants and germline variants of MRC5 coexisted within set B with the replacement.

A similar post-filtration performed for set A was applied to set B. First, sequencing coverage filtration was equally applied. Second, the VAF in each germline variant site was assessed to filter out sites that violate beta-binomial distribution for heterozygote [74, 76 for α, β calculated from MRC5 heterozygous single-nucleotide polymorphisms (SNPs), two tailed, p < 0.01] and homozygote (VAF < 0.9) to consider over-dispersion and capture bias in WES. Lastly, variant coverage was calculated to remove germline variants that were missing in any of mixture samples (variant coverage < 1).

## Data Records

The raw WES FASTQ files of 6 cell lines and 39 mixtures are available from the Sequence Read Archive under the accession code [PRJNA758606]^45^. Thirty-nine pairs of set A and set B are also available in BAM file format to be readily applied for evaluation of methods. Positive and negative controls of mosaic reference standards are available in GitHub^46^. The expected VAFs and compositions of positive controls in each sample are presented in Table 2.

## Technical Validation

### Validation of normal cell line stability

**Fig. 2:**
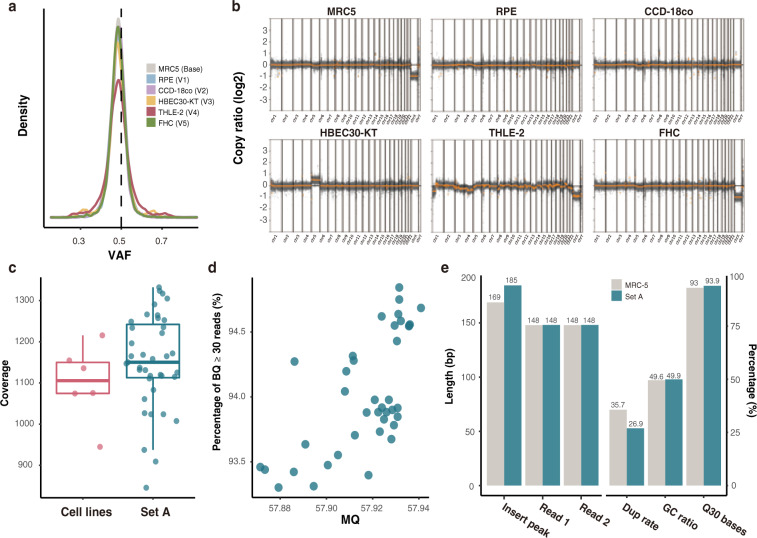
Quality validation for materials and sequencing data. (**a**) Distribution of heterozygous SNPs of the six cell lines. (**b**) Copy number ratio of the six cell lines. (**c**) Sequence coverage of the 6 cell lines and 39 mixtures of Set A. (**d**) Distribution of mean MQ and the percentage of bases with BQ over 30 in Set A. (**e**) Comparison of the read features between WES data of MRC-5 and mean values of 39 Set A. The lengths of insert size peak, paired read 1, paired read 2 and the percentages of PCR duplication rate (Dup rate), GC ratio, proportion of high-quality bases (BQ30) were compared. SNP single nucleotide polymorphism, MQ mapping quality, BQ base quality, WES whole exome sequencing.

We used six normal immortalized cell lines for stability and reproducibility, as they do not continuously acquire small and large variants during cell culture, unlike cancer cell lines. The distribution of heterozygous SNPs detected using Strelka2 annotated with gnomAD (v2.1.1) showed a singular peak at VAF 0.5 in all six cell lines, demonstrating the monoclonality of the materials (Fig. 2a). As positive controls were constructed by mixing independent cell lines, it was important to validate their diploid genotypes. Therefore, the overall regions of all six cell lines appeared to be copy number neutral, except the sex chromosomes and entire chromosome 5 of HBEC30-KT, as commonly observed^47^ (Fig. 2b). The unique germline variants used for the positive control were selected from copy number neutral regions through CNV analysis (Methods).

### Sequencing quality validation

We validated 45 WES data generated in this study, including the sequencing reads of 6 cell lines and 39 mixtures. We calculated the percentage of bases with phred-scaled base quality over 30, establishing an average value of 93.93% and a minimum of 91.82% among all data. The average GC content was 49.87%, with a maximum of 51.27%, thereby depicting a very low rate of bias during library preparation. FastQC and Qualimap were also applied to validate multiple quality of sequenced reads. Sequence quality of bases in read ends had steadily high base quality over 30. Data of both cell lines and mixtures showed high coverage, with more than 1,100× on average (Fig. 2c). We provided WES data with high coverage and quality for cell lines as well as set A to collect reliable germline variants and remove somatic variants with high VAF, which could serve as confounding factors when selected as positive controls. The mean mapping quality and base percentage with high-quality (BQ ≥ 30) of set A are shown in Fig. 2d. We also compared multiple features of reads from 39 set A and MRC5 data, which were merged when generating Set B. However, no significant differences were found, inferring that set B is less likely to have bias of two different sources (Fig. 2e).

**Fig. 3:**
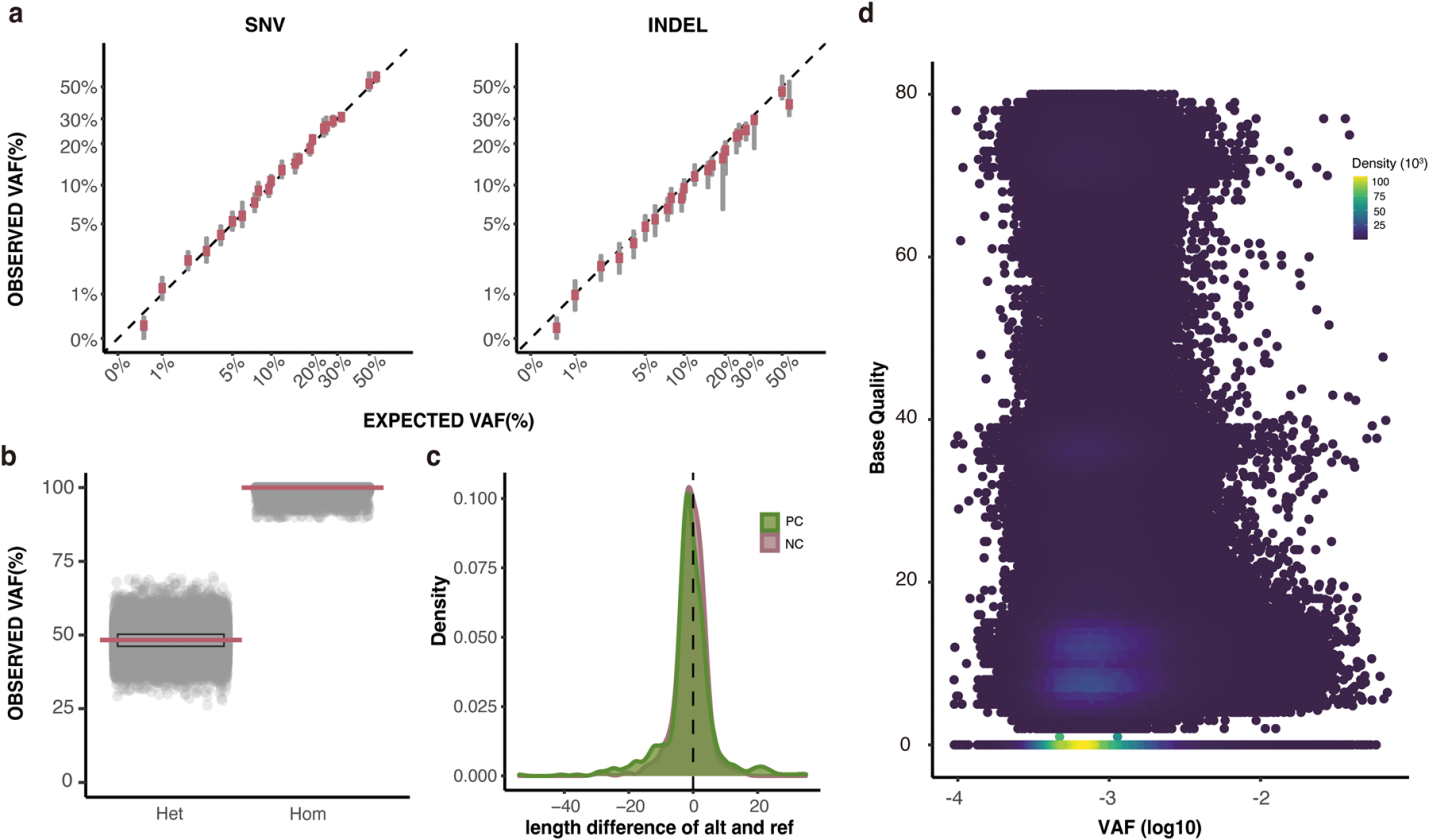
Quality validation of positive and negative controls. (**a**) Correlations of expected and observed VAF of positive SNVs (r = 0.97, p < 2.2e-16) and INDELs (r = 0.90, p < 2.2e-16) are shown in log 10 scale. Red lines: median VAF of observed VAFs. (**b**) VAF distributions of germline negative controls. (**c**) Length difference between alternative allele and reference allele of PC INDELs and NC germline INDELs. (**d**) Base qualities and log 10 transformed VAFs of artifacts in chromosome 1 of the random sample M2-5. The density of qualitative and quantitative distribution of artifacts were calculated from unexpected alternative alleles in Set A non-variant negative controls and depicted using ggpointdensity. VAF variant allele frequency, Het heterozygous, Hom homozygous, PC positive control, NC negative control.

### Quality Validation of positive and negative control

First, to validate the quality of positive controls, we investigated the correlations between expected VAFs of the design and observed VAFs in set A. Both SNVs and INDELs in the entire range of VAFs had a high coefficient of Pearson correlation between expected VAFs and the median value of observed VAFs among all positions with the same expected VAF (r = 0.97, p < 2.2e-16 and r = 0.91, p < 2.2e-16, respectively, shown in log10 scale in Fig. 3a). In other words, secure collection of germline variants (utilized as positive controls) within high coverage data (1,100×) could eliminate the possible ambiguity in the reference data, which can be originated from sub-clonal mutations acquired during cell culture. Thereafter, we assessed the distribution of germline negative controls in set B. The distribution of heterozygous and homozygous SNPs and INDELs is shown in Fig. 3b. The length of INDELs in positive controls and germline negative controls demonstrated a similar distribution, indicating that they could be comparably adjusted to variant callers for performance evaluation (Fig. 3c). The count of INDELs displayed a resemblance between them and most had a length smaller than 5 base pairs. Finally, we identified the quantitative and qualitative aspects of non-variant negative controls in set A. The raw alternative alleles were counted using SAMtools mplileup.

It was noteworthy that approximately one-third of the total target positions (10,202,428 in median of 39 reference data) were found to have more than one unexpected alternative allele in the non-variant positions (negative control of set A), in our ultra-high depth data (1,100×). In other words, abundant artifacts, unexpected alternative alleles produced during sequencing process, could have been generated owing to the advantage of multiple independent high coverage sequencing of the biological reference standards. Since detecting mosaic variants with low allele frequencies is extremely challenging, investigating those sites containing various read features would yield meaningful information for their accurate detection. For instance, in Fig. 3d, we demonstrated those sites within the chromosome 1 of the randomly selected sample (M2–5) with their base qualities and VAFs. They had a wide range of base quality, from 0 to 80, and artifacts were concentrated at VAF near 0.001, with a base quality of zero. However, a notable number of artifacts was found with high base quality, and the destructive effect of these artifacts is assumed to be greater in data with low-sequencing depth.

## Usage Notes

Each pair of reference data, namely, set A and set B, can be applied to detection methods and the resultant variant calls and their properties can be assessed via a comparison to the list of positive and negative controls provided in GitHub^46^. Evaluation of the true positive calls as well as both types of false positives based on two-types of negative controls, artifacts from set A and germline variant from set B, is possible. We recommend exploiting abundant number of provided reference data for robust evaluation. Although remarkable amount of mosaic variants with varied VAFs (especially lower than 10%) could be provided by means of cell line mixture-based reference standards, each data contains variants in limited number of expected VAFs (e.g., M1-1 has mosaic variants in four expected allele frequencies, 1%, 2%, 4%, and 8%). Hence, data selection with unbiased VAF distribution for their application is essential. The variant compositions as well as allele frequencies of the complete set of samples are shown in Table 2.

The provided reference data can be utilized for versatile analyses for mosaicism detection. For example, down-sampling of ultra-deep WES data (1,100×) will unveil detection accuracy in the lower depth of interest, yielding the information of how the sequencing coverage affect the performance of given methods. Also, variants with diversified VAFs in the provided data would support to reveal the thresholds of sequencing coverage for detecting low VAF variants. Also, accuracy of shared and sample-specific mosaic variant detection can be assessed under varied inter-sample VAF relationships. The reference data provides chances to evaluate and develop new detection algorithms for shared and sample-specific variants. For instance, thirty-nine reference dataset provide chance to assess up to 741 combinations by selecting two samples. Likewise, shared variant analysis among more than three samples are possible in even larger number of cases. Confident set of controls supports robust evaluations, and consequently, the reference data provides valuable opportunities for analyzing various aspects that should be considered in mosaic variant calling.

## Data Availability

The scripts used for constructing reference standards are available in a public repository GitHub^46^ (https://github.com/Yonsei-TGIL/Mosaic-Reference-Standards.git) and are accompanied by markdowns for a step-by-step description.

## Online-only Tables

**Online-only Table 1:** Cell line authentication by short tandem repeat markers.

Cell line	Reference match	Source	STR marker																	
			D5S818	D13S317	D7S820	D16S539	vWA	TH01	TPOX	CSF1PO	Amelogenin	D3S1358	D21S11	D18S51	D8S1179	FGA	D2S1338	D19S433	Penta D	Penta E
**CCD-18co**	84.6%	Used	11, 12	8, 12, 13, 13.3	8, 12	9, 10, 11, 12	16, 17, 18	6, 7, 9	8, 11	8, 12	X	15, 16, 17, 18	28.2, 29, 30	12, 17, 19	13, 14	18, 21, 24, 28	17, 19, 22	12.2, 13, 14	8, 9	7, 12
		Reference	12	12	8	12, 13	15, 17	6, 7	8, 11	8	X									
**FHC**	94.1%	Used	9, 11, 12	8, 12, 13	8, 9, 12	9, 10, 11	16, 17	6, 7, 9, 9.3	8, 11	11, 12	X, Y	15, 16, 17, 18	28, 29	15, 17	13, 14, 17	21, 24.3	17, 20	13, 14.2	9, 13	7, 14
		Reference	12, 13	12, 13	8, 12	9, 11	16	6, 9.3	8, 11	11, 12	X, Y									
**HBECO30-KT**	100%	Used	11, 12	8, 11, 12	8, 9	9, 10, 11	16, 17	6, 7, 9, 9.3	8, 9, 11	10, 11	X	15, 16, 17	28, 30	14, 16	13, 14	20	20, 25	12, 16.2	9, 13	10
		Reference	11	11, 12	8, 9	11	16, 17	9.3	8, 9	10, 11	X									
**MRC5**	100%	Used	9, 11, 12	8, 11, 14	8, 10, 11, 12	9, 10, 11	15, 16, 17, 18	6, 7, 8, 9	8, 11	11, 12	X, Y	15, 17	29, 31.2	12, 15, 21	13, 14	21, 23	17, 20	14, 15	12	12, 16
		Reference	11, 12	11, 14	10, 11	9, 11	15	8	8	11, 12	X, Y									
**RPE**	NA	Used	11, 12	8, 13	8, 10, 12	9, 10, 11, 13	16, 17, 18	6, 7, 9	8, 11	11, 12	X	15, 17, 18	28, 29, 30	12, 14	13, 14, 15	21, 22, 23	17, 23	13, 14.2	9, 10, 11	13, 14
		Reference	NA	NA	NA	NA	NA	NA	NA	NA	NA	NA	NA	NA	NA	NA	NA	NA	NA	NA
**THLE-2**	100%	Used	11, 13	8, 12	10, 12	9, 10, 11, 13	16, 17	6, 7, 9, 9.3	8, 11	11, 13	X, Y	14, 15	30, 32.2	13, 16	13	22, 24	17, 23	14	10, 13	12, 13
		Reference	11, 13	8, 12	10, 12	11, 13	16, 17	7, 9.3	8, 11	11, 13	X, Y									

Profiles of 17 short tandem repeat markers in used and reference database. Match percentage was calculated by dividing the number of shared alleles by total alleles in the reference database.

**Online-only Table 2:** Sequencing quality control matrix of cell lines and mixtures.

Cell lines	Median insert size	Mean mapping quality	Mean coverage	Library	Duplication rate	Insert size peak	Before Filtering (fastp)					After Filtering (fastp)					Filtering Results				
							Total reads (M)	Total bases (G)	Q20 bases (G)	Q30 bases (G)	GC content	Total reads (M)	Total bases (G)	Q20 bases (G)	Q30 bases (G)	GC content	Reads passed filters (M)	Reads with low quality (M)	Reads with too many N (K)	Reads too short (K)	Reads with low complexity (K)
**MRC5 (S0)**	209	57.32	1,135.66	Merged	35.67%	169	576.39	87.03	84.76 (97.38%)	80.95 (93.0%)	49.55%	568.98	84.33	82.46 (97.77%)	78.86 (93.51%)	49.40%	568.98 (98.71%)	5.73 (0.99%)	128.49 (0.02%)	1.36 (0.24%)	185.61 (0.03%)
**RPE (V1)**	213	57.30	944.90	Merged	31.76%	169	493.09	74.46	72.47 (97.34%)	69.05 (92.73%)	49.33%	487.34	72.24	70.59 (97.71%)	67.35 (93.22%)	49.20%	487.34 (98.83%)	4.67 (0.95%)	45.52 (0.01%)	925.43 (0.19%)	112.29 (0.02%)
**CCD-18co (V2)**	210	57.32	1,075.47	Merged	30.57%	173	538.84	81.37	79.25 (97.4%)	75.68 (93.01%)	49.40%	532.78	79.12	77.35 (97.76%)	73.98 (93.5%)	49.31%	532.78 (98.88%)	4.98 (0.92%)	119.85 (0.02%)	827.43 (0.15%)	129.28 (0.02%)
**HBEC30-KT (V3)**	209	57.33	1,154.61	Merged	43.08%	172	583	88.03	85.73 (97.38%)	81.78 (92.9%)	49.67%	577.14	85.56	83.62 (97.72%)	79.88 (93.35%)	49.56%	577.14 (99.0%)	4.84 (0.83%)	130.06 (0.02%)	759.35 (0.13%)	125.37 (0.02%)
**THLE-2 (V4)**	212	57.30	1,074.31	Merged	33.61%	178	546.67	82.55	80.46 (97.47%)	76.77 (93.0%)	49.16%	540.11	80.15	78.41 (97.83%)	74.92 (93.48%)	49.02%	540.11 (98.8%)	5.13 (0.94%)	52.11 (0.01%)	1.24 (0.23%)	148.99 (0.03%)
**FHC (V5)**	210	57.31	1,215.50	Merged	41.40%	169	617.41	93.23	91.26 (97.89%)	87.66 (94.03%)	49.44%	611.63	90.77	89.13 (98.19%)	85.72 (94.44%)	49.33%	611.63 (99.06%)	4.7 (0.76%)	107.1 (0.02%)	850.02 (0.14%)	128.41 (0.02%)
**Mixtures**	**Median insert size**	**Mean mapping quality**	**Mean coverage**	**Library**	**Duplication rate**	**Insert size peak**	**Before Filtering (fastp)**					**After Filtering (fastp)**					**Filtering Results**				
							**Total reads (M)**	**Total bases (G)**	**Q20 bases (G)**	**Q30 bases (G)**	**GC content**	**Total reads (M)**	**Total bases (G)**	**Q20 bases (G)**	**Q30 bases (G)**	**GC content**	**Reads passed filters (M)**	**Reads with low quality (M)**	**Reads with too many N (K)**	**Reads too short (K)**	**Reads with low complexity (K)**
**M1-1**	209	57.28	1,116.61	M1-1-1	21.64%	177	123.63	18.67	18.15 (97.23%)	17.29 (92.6%)	49.65%	121.93	18.13	17.7 (97.64%)	16.88 (93.14%)	49.55%	121.93 (98.62%)	1.41 (1.14%)	6.52 (0.01%)	253.86 (0.21%)	35.56 (0.03%)
				M1-1-2	20.93%	200	157.08	23.72	23.19 (97.75%)	22.26 (93.85%)	49.49%	155.65	23.23	22.78 (98.06%)	21.89 (94.24%)	49.39%	155.65 (99.09%)	1.22 (0.78%)	1.41 (0.0%)	177.15 (0.11%)	25.24 (0.02%)
				M1-1-3	22.38%	209	176.12	26.59	25.98 (97.7%)	24.93 (93.72%)	49.50%	174.48	26.06	25.54 (98.01%)	24.53 (94.12%)	49.41%	174.48 (99.07%)	1.4 (0.8%)	1.55 (0.0%)	199.77 (0.11%)	28.63 (0.02%)
				M1-1-4	18.37%	179	113.8	17.18	16.69 (97.15%)	15.9 (92.54%)	49.88%	111.88	16.63	16.24 (97.62%)	15.49 (93.15%)	49.76%	111.88 (98.31%)	1.52 (1.33%)	6.83 (0.01%)	361.91 (0.32%)	35.76 (0.03%)
**M1-2**	209	57.28	1,115.97	M1-2-1	23.63%	181	141.63	21.39	20.8 (97.24%)	19.81 (92.62%)	49.08%	139.76	20.75	20.26 (97.64%)	19.33 (93.15%)	48.96%	139.76 (98.68%)	1.56 (1.1%)	7.71 (0.01%)	266.4 (0.19%)	36.39 (0.03%)
				M1-2-2	22.92%	192	154.17	23.28	22.75 (97.73%)	21.85 (93.84%)	49.81%	152.67	22.75	22.31 (98.07%)	21.45 (94.27%)	49.71%	152.67 (99.03%)	1.28 (0.83%)	1.51 (0.0%)	191.36 (0.12%)	26.4 (0.02%)
				M1-2-3	20.27%	200	150.14	22.67	21.96 (96.87%)	20.85 (91.95%)	49.70%	148.12	22.08	21.48 (97.31%)	20.42 (92.51%)	49.58%	148.12 (98.66%)	1.74 (1.16%)	1.22 (0.0%)	255.89 (0.17%)	18.64 (0.01%)
				M1-2-4	23.04%	176	298.6	45.09	43.63 (96.76%)	41.29 (91.58%)	49.54%	293.79	43.62	42.41 (97.22%)	40.21 (92.18%)	49.41%	293.79 (98.39%)	3.78 (1.27%)	10.86 (0.0%)	895.06 (0.3%)	122.1 (0.04%)
**M1-3**	209	57.28	1,117.94	M1-3-1	25.79%	184	121.61	18.36	17.83 (97.1%)	16.96 (92.35%)	49.84%	119.89	17.83	17.39 (97.53%)	16.56 (92.91%)	49.74%	119.89 (98.59%)	1.46 (1.2%)	6.57 (0.01%)	221.39 (0.18%)	29.54 (0.02%)
				M1-3-2	19.76%	206	126.35	19.08	18.64 (97.72%)	17.9 (93.82%)	49.54%	125.09	18.66	18.3 (98.06%)	17.59 (94.25%)	49.44%	125.09 (99.01%)	1.07 (0.85%)	1.1 (0.0%)	162.93 (0.13%)	22.94 (0.02%)
				M1-3-3	28.51%	199	316.59	47.8	46.18 (96.6%)	43.57 (91.14%)	49.46%	312.52	46.61	45.21 (96.99%)	42.72 (91.65%)	49.37%	312.52 (98.72%)	3.53 (1.11%)	15.27 (0.0%)	458.77 (0.14%)	64.22 (0.02%)
				M1-3-4	15.93%	175	119.01	17.97	17.45 (97.13%)	16.62 (92.46%)	49.43%	117.12	17.4	16.98 (97.59%)	16.19 (93.06%)	49.32%	117.12 (98.41%)	1.52 (1.28%)	7.31 (0.01%)	326.55 (0.27%)	31.71 (0.03%)
**M1-4**	209	57.28	1,116.68	M1-4-1	21.91%	178	107.41	16.22	15.76 (97.16%)	15.0 (92.47%)	50.16%	105.97	15.77	15.38 (97.57%)	14.66 (93.01%)	50.06%	105.97 (98.65%)	1.23 (1.14%)	5.73 (0.01%)	185.66 (0.17%)	24.23 (0.02%)
				M1-4-2	22.47%	191	102.38	15.46	15.14 (97.91%)	14.58 (94.29%)	49.68%	101.42	15.1	14.83 (98.24%)	14.3 (94.71%)	49.56%	101.42 (99.06%)	789.32 (0.77%)	1.86 (0.0%)	155.38 (0.15%)	20.24 (0.02%)
				M1-4-3	36.61%	192	304.65	46	44.91 (97.62%)	43.0 (93.47%)	49.81%	301.68	44.88	43.96 (97.94%)	42.14 (93.88%)	49.72%	301.68 (99.03%)	2.43 (0.8%)	3.49 (0.0%)	438.73 (0.14%)	87.92 (0.03%)
				M1-4-4	21.27%	170	130.14	19.65	19.13 (97.34%)	18.27 (93.0%)	49.60%	128.18	18.99	18.57 (97.81%)	17.77 (93.61%)	49.46%	128.18 (98.5%)	1.57 (1.21%)	7.49 (0.01%)	340.6 (0.26%)	35.34 (0.03%)
**M1-5**	209	57.28	1,116.16	M1-5-1	23.30%	182	121.26	18.31	17.78 (97.12%)	16.91 (92.36%)	50.15%	119.64	17.79	17.35 (97.53%)	16.53 (92.9%)	50.05%	119.64 (98.66%)	1.38 (1.14%)	6.45 (0.01%)	211.38 (0.17%)	25.87 (0.02%)
				M1-5-2	25.75%	189	152.32	23	22.44 (97.58%)	21.52 (93.55%)	50.03%	150.68	22.42	21.96 (97.96%)	21.08 (94.03%)	49.91%	150.68 (98.92%)	1.38 (0.91%)	1.41 (0.0%)	232.2 (0.15%)	27.77 (0.02%)
				M1-5-3	24.30%	181	142.22	21.48	21.01 (97.81%)	20.21 (94.1%)	50.04%	140.76	20.91	20.53 (98.18%)	19.77 (94.57%)	49.92%	140.76 (98.97%)	1.19 (0.84%)	1.19 (0.0%)	232.47 (0.16%)	31.89 (0.02%)
				M1-5-4	20.14%	179	143.58	21.68	21.05 (97.11%)	20.04 (92.43%)	49.95%	141.24	20.97	20.47 (97.59%)	19.52 (93.06%)	49.84%	141.24 (98.37%)	1.88 (1.31%)	8.82 (0.01%)	415.17 (0.29%)	40.75 (0.03%)
**M1-6**	209	57.28	1,113.98	M1-6-1	24.30%	190	114.9	17.35	16.88 (97.31%)	16.1 (92.77%)	49.99%	113.5	16.88	16.49 (97.69%)	15.74 (93.27%)	49.90%	113.5 (98.78%)	1.19 (1.04%)	6.1 (0.01%)	175.86 (0.15%)	24.96 (0.02%)
				M1-6-2	19.99%	180	103.76	15.67	15.33 (97.84%)	14.74 (94.08%)	49.63%	102.86	15.3	15.02 (98.16%)	14.46 (94.49%)	49.53%	102.86 (99.13%)	781.96 (0.75%)	898.0 (0.0%)	101.57 (0.1%)	16.2 (0.02%)
				M1-6-3	22.09%	178	150.6	22.74	22.25 (97.83%)	21.4 (94.09%)	50.07%	149.3	22.19	21.78 (98.17%)	20.97 (94.51%)	49.97%	149.3 (99.14%)	1.13 (0.75%)	1.32 (0.0%)	149.59 (0.1%)	22.98 (0.02%)
				M1-6-4	22.06%	176	305.96	46.2	44.88 (97.14%)	42.67 (92.36%)	49.99%	302.51	44.85	43.73 (97.5%)	41.64 (92.83%)	49.86%	302.51 (98.87%)	2.78 (0.91%)	7.92 (0.0%)	590.16 (0.19%)	69.98 (0.02%)
**M1-7**	209	57.28	1,114.57	M1-7-1	26.51%	174	146.8	22.17	21.5 (97.0%)	20.54 (92.66%)	50.25%	144.37	21.43	20.92 (97.6%)	20.03 (93.44%)	50.13%	144.37 (98.35%)	2.12 (1.44%)	3.45 (0.0%)	260.95 (0.18%)	46.25 (0.03%)
				M1-7-2	18.50%	174	109.79	16.58	16.22 (97.86%)	15.61 (94.16%)	49.39%	108.83	16.16	15.87 (98.2%)	15.29 (94.6%)	49.26%	108.83 (99.12%)	822.37 (0.75%)	978.0 (0.0%)	123.96 (0.11%)	19.05 (0.02%)
				M1-7-3	22.67%	190	147.3	22.24	21.75 (97.77%)	20.89 (93.93%)	49.73%	145.99	21.74	21.32 (98.1%)	20.51 (94.35%)	49.63%	145.99 (99.11%)	1.14 (0.78%)	1.35 (0.0%)	146.98 (0.1%)	20.62 (0.01%)
				M1-7-4	22.92%	170	277.07	41.84	40.7 (97.28%)	38.78 (92.7%)	50.39%	274.07	40.63	39.67 (97.63%)	37.85 (93.16%)	50.28%	274.07 (98.92%)	2.39 (0.86%)	7.89 (0.0%)	526.79 (0.19%)	66.79 (0.02%)
**M1-8**	209	57.28	1,115.07	M1-8-1	22.16%	171	133	20.08	19.48 (96.99%)	18.6 (92.62%)	49.91%	130.82	19.41	18.94 (97.6%)	18.13 (93.42%)	49.78%	130.82 (98.36%)	1.9 (1.43%)	3.02 (0.0%)	232.44 (0.17%)	41.74 (0.03%)
				M1-8-2	21.55%	197	130.64	19.73	19.3 (97.83%)	18.55 (94.04%)	49.84%	129.52	19.3	18.94 (98.14%)	18.23 (94.43%)	49.75%	129.52 (99.14%)	972.16 (0.74%)	1.2 (0.0%)	127.51 (0.1%)	19.64 (0.02%)
				M1-8-3	22.66%	190	138.71	20.95	20.5 (97.89%)	19.73 (94.2%)	49.83%	137.52	20.48	20.11 (98.2%)	19.37 (94.6%)	49.74%	137.52 (99.14%)	1.02 (0.74%)	1.24 (0.0%)	143.37 (0.1%)	23.51 (0.02%)
				M1-8-4	24.07%	173	278.18	42	40.91 (97.38%)	39.05 (92.97%)	50.28%	274.75	40.77	39.85 (97.76%)	38.1 (93.46%)	50.16%	274.75 (98.77%)	2.59 (0.93%)	7.35 (0.0%)	730.72 (0.26%)	91.63 (0.03%)
**M1-9**	209	57.28	1,116.31	M1-9-1	41.25%	170	166.43	25.13	24.69 (98.24%)	23.9 (95.1%)	49.89%	164.66	24.43	24.09 (98.6%)	23.35 (95.57%)	49.74%	164.66 (98.93%)	1.41 (0.85%)	7.41 (0.0%)	315.19 (0.19%)	45.24 (0.03%)
				M1-9-2	24.90%	188	146.09	22.06	21.54 (97.66%)	20.68 (93.73%)	49.90%	144.46	21.52	21.09 (98.02%)	20.27 (94.19%)	49.79%	144.46 (98.88%)	1.33 (0.91%)	1.34 (0.0%)	269.62 (0.18%)	32.89 (0.02%)
				M1-9-3	24.28%	192	145.81	22.02	21.5 (97.67%)	20.64 (93.76%)	50.25%	144.25	21.48	21.05 (98.04%)	20.24 (94.23%)	50.14%	144.25 (98.93%)	1.29 (0.88%)	1.32 (0.0%)	240.99 (0.17%)	28.96 (0.02%)
				M1-9-4	33.88%	188	185.6	28.03	27.57 (98.36%)	26.72 (95.35%)	49.88%	183.4	27.27	26.92 (98.73%)	26.13 (95.82%)	49.71%	183.4 (98.82%)	1.55 (0.84%)	6.37 (0.0%)	577.3 (0.31%)	62.24 (0.03%)
**M2-1**	209	57.28	1,116.73	M2-1-1	41.67%	180	189.7	28.64	28.14 (98.24%)	27.25 (95.13%)	50.30%	187.58	27.85	27.47 (98.61%)	26.63 (95.61%)	50.17%	187.58 (98.88%)	1.65 (0.87%)	8.55 (0.0%)	404.57 (0.21%)	56.14 (0.03%)
				M2-1-2	20.97%	171	109.44	16.53	16.17 (97.82%)	15.56 (94.14%)	50.71%	108.34	16.05	15.77 (98.2%)	15.19 (94.63%)	50.59%	108.34 (98.99%)	912.67 (0.83%)	934.0 (0.0%)	166.98 (0.15%)	23.22 (0.02%)
				M2-1-3	25.16%	172	190.8	28.81	28.18 (97.81%)	27.11 (94.11%)	49.58%	188.96	27.98	27.47 (98.19%)	26.46 (94.6%)	49.43%	188.96 (99.03%)	1.53 (0.8%)	1.7 (0.0%)	277.67 (0.15%)	37.94 (0.02%)
				M2-1-4	32.93%	186	163.27	24.65	24.26 (98.39%)	23.53 (95.43%)	49.65%	161.28	23.99	23.69 (98.76%)	23.01 (95.9%)	49.49%	161.28 (98.78%)	1.44 (0.88%)	5.38 (0.0%)	477.85 (0.29%)	62.87 (0.04%)
**M2-2**	209	57.28	1,117.05	M2-2-1	36.19%	184	175.76	26.54	26.07 (98.22%)	25.22 (95.02%)	50.09%	173.96	25.86	25.49 (98.57%)	24.69 (95.47%)	49.96%	173.96 (98.97%)	1.44 (0.82%)	7.96 (0.0%)	313.57 (0.18%)	42.37 (0.02%)
				M2-2-2	22.07%	185	128.54	19.41	18.99 (97.84%)	18.27 (94.13%)	50.07%	127.32	18.9	18.55 (98.19%)	17.87 (94.59%)	49.95%	127.32 (99.05%)	1.02 (0.79%)	1.13 (0.0%)	175.16 (0.14%)	23.96 (0.02%)
				M2-2-3	22.34%	181	140.4	21.2	20.74 (97.81%)	19.94 (94.06%)	49.63%	139.15	20.66	20.28 (98.15%)	19.53 (94.51%)	49.52%	139.15 (99.11%)	1.07 (0.76%)	1.29 (0.0%)	148.55 (0.11%)	23.32 (0.02%)
				M2-2-4	25.77%	180	179.06	27.04	26.37 (97.54%)	25.23 (93.3%)	49.70%	176.57	26.23	25.69 (97.94%)	24.61 (93.81%)	49.57%	176.57 (98.61%)	1.91 (1.07%)	7.82 (0.0%)	526.01 (0.29%)	51.07 (0.03%)
**M2-3**	209	57.28	1,116.35	M2-3-1	32.82%	180	159.01	24.01	23.57 (98.15%)	22.76 (94.81%)	49.99%	157.41	23.42	23.07 (98.49%)	22.31 (95.25%)	49.87%	157.41 (98.99%)	1.29 (0.81%)	6.94 (0.0%)	277.37 (0.17%)	31.22 (0.02%)
				M2-3-2	26.94%	178	165.24	24.95	24.4 (97.79%)	23.47 (94.06%)	50.19%	163.57	24.25	23.8 (98.17%)	22.92 (94.55%)	50.06%	163.57 (98.99%)	1.38 (0.84%)	1.36 (0.0%)	253.35 (0.15%)	35.22 (0.02%)
				M2-3-3	20.48%	176	98.52	14.88	14.55 (97.8%)	13.99 (94.04%)	49.40%	97.55	14.46	14.2 (98.17%)	13.67 (94.52%)	49.25%	97.55 (99.02%)	793.29 (0.81%)	870.0 (0.0%)	149.57 (0.15%)	21.28 (0.02%)
				M2-3-4	27.71%	187	166.42	25.13	24.52 (97.56%)	23.45 (93.33%)	49.90%	164.13	24.4	23.9 (97.95%)	22.9 (93.84%)	49.77%	164.13 (98.62%)	1.75 (1.05%)	7.17 (0.0%)	481.54 (0.29%)	49.2 (0.03%)
**M2-4**	209	57.28	1,116.65	M2-4-1	37.14%	185	161.52	24.39	23.96 (98.25%)	23.2 (95.12%)	50.34%	159.9	23.76	23.43 (98.6%)	22.71 (95.58%)	50.21%	159.9 (99.0%)	1.29 (0.8%)	7.16 (0.0%)	280.75 (0.17%)	36.77 (0.02%)
				M2-4-2	26.11%	186	161.41	24.37	23.8 (97.66%)	22.81 (93.58%)	49.74%	159.22	23.65	23.18 (98.05%)	22.25 (94.08%)	49.61%	159.22 (98.64%)	1.66 (1.03%)	6.96 (0.0%)	475.77 (0.29%)	51.68 (0.03%)
				M2-4-3	25.86%	234	132.35	19.98	19.53 (97.71%)	18.74 (93.78%)	49.64%	130.63	19.68	19.29 (98.03%)	18.53 (94.18%)	49.55%	130.63 (98.7%)	1.32 (1.0%)	1.36 (0.0%)	357.12 (0.27%)	39.0 (0.03%)
				M2-4-4	25.92%	231	171.69	25.93	25.36 (97.82%)	24.38 (94.06%)	49.65%	169.46	25.52	25.05 (98.14%)	24.11 (94.45%)	49.56%	169.46 (98.7%)	1.69 (0.98%)	1.57 (0.0%)	489.98 (0.29%)	58.21 (0.03%)
**M2-5**	209	57.28	1,115.66	M2-5-1	35.91%	180	141.48	21.36	20.97 (98.14%)	20.25 (94.79%)	50.20%	140.01	20.81	20.5 (98.49%)	19.82 (95.24%)	50.07%	140.01 (98.96%)	1.17 (0.83%)	6.33 (0.0%)	264.51 (0.19%)	30.38 (0.02%)
				M2-5-2	29.27%	229	119.32	18.02	17.64 (97.91%)	16.98 (94.24%)	49.86%	117.88	17.75	17.43 (98.21%)	16.79 (94.62%)	49.78%	117.88 (98.79%)	1.11 (0.93%)	1.16 (0.0%)	293.51 (0.25%)	36.63 (0.03%)
				M2-5-3	26.41%	230	174.04	26.28	25.66 (97.65%)	24.61 (93.66%)	49.67%	171.77	25.87	25.34 (97.98%)	24.33 (94.07%)	49.58%	171.77 (98.7%)	1.76 (1.01%)	1.63 (0.0%)	460.85 (0.26%)	45.74 (0.03%)
				M2-5-4	25.26%	180	139.39	21.05	20.5 (97.38%)	19.59 (93.08%)	49.51%	137.14	20.35	19.91 (97.85%)	19.07 (93.69%)	49.36%	137.14 (98.38%)	1.73 (1.24%)	7.81 (0.01%)	467.47 (0.34%)	47.71 (0.03%)
**M2-6**	209	57.28	1,116.73	M2-6-1	40.30%	184	183.9	27.77	27.28 (98.26%)	26.41 (95.1%)	50.43%	182.07	27.09	26.71 (98.59%)	25.88 (95.53%)	50.31%	182.07 (99.01%)	1.45 (0.79%)	8.49 (0.0%)	318.81 (0.17%)	41.79 (0.02%)
				M2-6-2	29.73%	235	172.85	26.1	25.53 (97.82%)	24.54 (94.04%)	50.00%	170.76	25.71	25.23 (98.12%)	24.28 (94.42%)	49.92%	170.76 (98.79%)	1.64 (0.95%)	1.6 (0.0%)	398.86 (0.23%)	48.38 (0.03%)
				M2-6-3	23.56%	232	136	20.54	20.07 (97.72%)	19.26 (93.8%)	49.81%	134.32	20.23	19.83 (98.03%)	19.06 (94.19%)	49.72%	134.32 (98.76%)	1.32 (0.97%)	1.29 (0.0%)	325.79 (0.24%)	35.32 (0.03%)
				M2-6-4	18.52%	176	137.62	20.78	20.16 (97.02%)	19.17 (92.27%)	49.37%	135.19	20.06	19.56 (97.54%)	18.64 (92.94%)	49.24%	135.19 (98.23%)	1.95 (1.41%)	8.25 (0.01%)	436.38 (0.32%)	42.92 (0.03%)
**M2-7**	209	57.28	1,115.50	M2-7-1	33.66%	174	135.03	20.39	20.04 (98.26%)	19.4 (95.13%)	50.09%	133.73	19.84	19.57 (98.61%)	18.97 (95.58%)	49.95%	133.73 (99.04%)	1.04 (0.77%)	5.99 (0.0%)	225.16 (0.17%)	30.63 (0.02%)
				M2-7-2	21.67%	232	108.77	16.42	16.07 (97.86%)	15.45 (94.09%)	49.86%	107.47	16.19	15.89 (98.15%)	15.29 (94.46%)	49.78%	107.47 (98.8%)	1.01 (0.93%)	1.01 (0.0%)	261.24 (0.24%)	31.65 (0.03%)
				M2-7-3	25.80%	232	134.55	20.32	19.85 (97.68%)	19.04 (93.73%)	49.56%	132.78	20	19.6 (98.01%)	18.83 (94.13%)	49.47%	132.78 (98.69%)	1.36 (1.01%)	1.36 (0.0%)	369.01 (0.27%)	38.21 (0.03%)
				M2-7-4	17.42%	189	147.68	22.3	21.63 (96.98%)	20.56 (92.19%)	49.79%	144.97	21.54	21.0 (97.51%)	20.0 (92.87%)	49.67%	144.97 (98.16%)	2.18 (1.48%)	9.23 (0.01%)	480.13 (0.33%)	47.29 (0.03%)
**M2-8**	209	57.28	1,114.94	M2-8-1	41.11%	182	144.95	21.89	21.5 (98.23%)	20.8 (95.04%)	50.32%	143.48	21.34	21.04 (98.57%)	20.38 (95.49%)	50.19%	143.48 (98.99%)	1.17 (0.81%)	6.62 (0.0%)	258.85 (0.18%)	32.99 (0.02%)
				M2-8-2	29.36%	168	125.62	18.97	18.55 (97.8%)	17.84 (94.07%)	49.97%	124.31	18.42	18.08 (98.18%)	17.42 (94.57%)	49.84%	124.31 (98.96%)	1.07 (0.85%)	1.2 (0.0%)	214.22 (0.17%)	29.81 (0.02%)
				M2-8-3	23.03%	182	124.46	18.79	18.37 (97.74%)	17.65 (93.91%)	49.54%	123.22	18.3	17.96 (98.1%)	17.28 (94.38%)	49.41%	123.22 (99.0%)	1.04 (0.83%)	1.07 (0.0%)	175.64 (0.14%)	24.74 (0.02%)
				M2-8-4	27.72%	181	139.98	21.14	20.46 (96.8%)	19.41 (91.82%)	49.79%	137.23	20.39	19.85 (97.35%)	18.87 (92.53%)	49.65%	137.23 (98.04%)	2.19 (1.57%)	8.64 (0.01%)	500.51 (0.36%)	43.25 (0.03%)
**M2-9**	209	57.28	1,115.88	M2-9-1	42.19%	179	154.43	23.32	22.91 (98.25%)	22.16 (95.04%)	50.40%	153.02	22.77	22.45 (98.57%)	21.74 (95.45%)	50.28%	153.02 (99.08%)	1.17 (0.76%)	6.92 (0.0%)	206.22 (0.13%)	28.18 (0.02%)
				M2-9-2	20.34%	179	108.43	16.37	16.02 (97.82%)	15.4 (94.05%)	49.89%	107.49	15.97	15.67 (98.16%)	15.09 (94.48%)	49.78%	107.49 (99.13%)	822.63 (0.76%)	982.0 (0.0%)	104.27 (0.1%)	16.54 (0.02%)
				M2-9-3	25.64%	179	166.75	25.18	24.59 (97.65%)	23.59 (93.68%)	49.95%	165.25	24.52	24.03 (98.01%)	23.09 (94.14%)	49.83%	165.25 (99.1%)	1.33 (0.8%)	1.56 (0.0%)	146.24 (0.09%)	20.98 (0.01%)
				M2-9-4	17.63%	183	133.09	20.1	19.47 (96.9%)	18.49 (91.99%)	49.76%	130.77	19.46	18.96 (97.41%)	18.03 (92.65%)	49.66%	130.77 (98.26%)	1.93 (1.45%)	8.06 (0.01%)	350.17 (0.26%)	31.81 (0.02%)
**M2-10**	209	57.28	1,117.66	M2-10-1	33.19%	181	134.71	20.34	19.97 (98.2%)	19.31 (94.93%)	50.39%	133.43	19.86	19.56 (98.53%)	18.93 (95.35%)	50.27%	133.43 (99.05%)	1.05 (0.78%)	6.16 (0.0%)	191.35 (0.14%)	25.07 (0.02%)
				M2-10-2	22.60%	175	182.3	27.53	26.89 (97.69%)	25.81 (93.76%)	49.64%	180.69	26.84	26.31 (98.04%)	25.28 (94.2%)	49.53%	180.69 (99.12%)	1.41 (0.78%)	1.62 (0.0%)	168.55 (0.09%)	24.27 (0.01%)
				M2-10-3	22.24%	184	156.49	23.63	23.13 (97.88%)	22.25 (94.18%)	49.95%	155.19	23.09	22.67 (98.19%)	21.84 (94.59%)	49.85%	155.19 (99.17%)	1.14 (0.73%)	1.45 (0.0%)	133.57 (0.09%)	23.74 (0.02%)
				M2-10-4	34.17%	184	196.39	29.66	29.2 (98.45%)	28.34 (95.57%)	50.48%	194.27	28.89	28.53 (98.79%)	27.73 (96.0%)	50.34%	194.27 (98.92%)	1.51 (0.77%)	6.65 (0.0%)	543.75 (0.28%)	59.42 (0.03%)
**M2-11**	209	57.28	1,116.69	M2-11-1	38.97%	179	187.5	28.31	27.82 (98.26%)	26.92 (95.07%)	49.73%	185.77	27.61	27.22 (98.59%)	26.37 (95.5%)	49.59%	185.77 (99.08%)	1.42 (0.76%)	8.39 (0.0%)	262.0 (0.14%)	37.36 (0.02%)
				M2-11-2	20.65%	188	125.43	18.94	18.51 (97.75%)	17.78 (93.86%)	49.56%	124.35	18.51	18.15 (98.07%)	17.45 (94.27%)	49.46%	124.35 (99.14%)	953.65 (0.76%)	1.18 (0.0%)	110.88 (0.09%)	17.13 (0.01%)
				M2-11-3	20.95%	182	136.31	20.58	20.15 (97.89%)	19.39 (94.21%)	49.81%	135.17	20.07	19.71 (98.21%)	18.99 (94.63%)	49.70%	135.17 (99.16%)	988.14 (0.72%)	1.17 (0.0%)	127.69 (0.09%)	22.06 (0.02%)
				M2-11-4	35.42%	187	198.55	29.98	29.49 (98.36%)	28.57 (95.29%)	50.34%	196.44	29.25	28.86 (98.69%)	27.99 (95.72%)	50.20%	196.44 (98.93%)	1.55 (0.78%)	6.93 (0.0%)	509.8 (0.26%)	50.95 (0.03%)
**M2-12**	209	57.28	1,117.19	M2-12-1	36.36%	185	174.67	26.38	25.93 (98.3%)	25.11 (95.19%)	50.29%	173.08	25.73	25.38 (98.62%)	24.6 (95.61%)	50.18%	173.08 (99.09%)	1.31 (0.75%)	7.8 (0.0%)	240.9 (0.14%)	37.07 (0.02%)
				M2-12-2	21.18%	193	153.94	23.25	22.73 (97.8%)	21.85 (94.0%)	49.99%	152.54	22.72	22.29 (98.12%)	21.45 (94.42%)	49.90%	152.54 (99.09%)	1.22 (0.79%)	2.53 (0.0%)	158.13 (0.1%)	23.18 (0.02%)
				M2-12-3	23.33%	183	130	19.63	19.25 (98.08%)	18.59 (94.68%)	49.84%	128.96	19.17	18.86 (98.39%)	18.23 (95.08%)	49.73%	128.96 (99.19%)	885.16 (0.68%)	2.39 (0.0%)	135.64 (0.1%)	23.82 (0.02%)
				M2-12-4	37.49%	189	214.55	32.4	31.89 (98.43%)	30.94 (95.5%)	50.44%	212.21	31.59	31.2 (98.76%)	30.31 (95.93%)	50.30%	212.21 (98.91%)	1.72 (0.8%)	7.4 (0.0%)	551.74 (0.26%)	65.17 (0.03%)
**M3-1**	209	57.28	1,116.53	M3-1-1	36.29%	182	169.3	25.56	25.09 (98.16%)	24.24 (94.81%)	50.33%	167.74	24.96	24.58 (98.48%)	23.77 (95.23%)	50.22%	167.74 (99.08%)	1.29 (0.76%)	7.77 (0.0%)	227.31 (0.13%)	28.36 (0.02%)
				M3-1-2	19.84%	190	165.66	25.01	24.53 (98.06%)	23.67 (94.61%)	50.04%	164.33	24.45	24.05 (98.36%)	23.23 (95.0%)	49.95%	164.33 (99.2%)	1.12 (0.68%)	2.85 (0.0%)	172.02 (0.1%)	27.06 (0.02%)
				M3-1-3	18.75%	184	131.77	19.9	19.45 (97.74%)	18.67 (93.85%)	49.51%	130.56	19.41	19.04 (98.08%)	18.3 (94.3%)	49.40%	130.56 (99.09%)	1.05 (0.8%)	2.24 (0.0%)	135.12 (0.1%)	18.34 (0.01%)
				M3-1-4	33.08%	184	196.88	29.73	29.24 (98.36%)	28.33 (95.28%)	49.67%	194.69	28.97	28.59 (98.7%)	27.73 (95.71%)	49.52%	194.69 (98.89%)	1.55 (0.79%)	6.65 (0.0%)	582.79 (0.3%)	56.61 (0.03%)
**M3-2**	209	57.28	1,114.65	M3-2-1	35.02%	181	122.66	18.52	18.21 (98.29%)	17.63 (95.17%)	50.60%	121.59	18.09	17.84 (98.6%)	17.29 (95.57%)	50.49%	121.59 (99.13%)	886.75 (0.72%)	5.52 (0.0%)	156.4 (0.13%)	21.21 (0.02%)
				M3-2-2	19.35%	175	132.01	19.93	19.51 (97.89%)	18.78 (94.23%)	49.99%	130.82	19.4	19.06 (98.23%)	18.37 (94.68%)	49.86%	130.82 (99.1%)	1.01 (0.77%)	2.17 (0.0%)	154.69 (0.12%)	21.62 (0.02%)
				M3-2-3	20.27%	179	121.35	18.32	17.91 (97.72%)	17.2 (93.85%)	49.36%	120.2	17.84	17.49 (98.08%)	16.82 (94.32%)	49.22%	120.2 (99.05%)	981.04 (0.81%)	2.2 (0.0%)	152.95 (0.13%)	19.29 (0.02%)
				M3-2-4	32.90%	190	210.8	31.83	31.33 (98.44%)	30.4 (95.49%)	49.95%	208.62	31.1	30.71 (98.75%)	29.82 (95.89%)	49.81%	208.62 (98.97%)	1.56 (0.74%)	7.14 (0.0%)	554.01 (0.26%)	58.89 (0.03%)
**M3-3**	209	57.28	1,115.95	M3-3-1	32.05%	176	132.12	19.95	19.59 (98.2%)	18.95 (94.98%)	49.14%	130.84	19.41	19.13 (98.56%)	18.52 (95.44%)	48.97%	130.84 (99.03%)	1.03 (0.78%)	5.96 (0.0%)	221.08 (0.17%)	25.34 (0.02%)
				M3-3-2	35.19%	190	291.81	44.06	42.71 (96.93%)	40.49 (91.88%)	49.63%	288.36	42.97	41.81 (97.31%)	39.69 (92.38%)	49.57%	288.36 (98.82%)	2.99 (1.03%)	18.18 (0.01%)	356.77 (0.12%)	81.61 (0.03%)
				M3-3-3	21.60%	180	110.16	16.63	16.26 (97.78%)	15.63 (93.94%)	49.76%	109.14	16.24	15.93 (98.11%)	15.32 (94.37%)	49.65%	109.14 (99.08%)	872.52 (0.79%)	2.0 (0.0%)	125.49 (0.11%)	16.22 (0.01%)
				M3-3-4	35.92%	181	177.78	26.84	26.43 (98.46%)	25.66 (95.57%)	50.14%	175.85	26.14	25.83 (98.8%)	25.1 (96.0%)	49.99%	175.85 (98.92%)	1.36 (0.76%)	5.95 (0.0%)	504.05 (0.28%)	54.57 (0.03%)
**M3-4**	209	57.28	1,114.75	M3-4-1	31.85%	180	140.12	21.16	20.8 (98.29%)	20.14 (95.19%)	49.82%	138.81	20.62	20.34 (98.63%)	19.72 (95.62%)	49.70%	138.81 (99.06%)	1.08 (0.77%)	6.36 (0.0%)	199.83 (0.14%)	33.09 (0.02%)
				M3-4-2	24.73%	182	150.65	22.75	22.24 (97.75%)	21.36 (93.92%)	49.91%	149.21	22.18	21.76 (98.11%)	20.93 (94.38%)	49.79%	149.21 (99.04%)	1.23 (0.81%)	2.55 (0.0%)	189.59 (0.13%)	24.58 (0.02%)
				M3-4-3	22.09%	189	151.84	22.93	22.42 (97.77%)	21.54 (93.93%)	49.88%	150.37	22.37	21.95 (98.11%)	21.11 (94.37%)	49.76%	150.37 (99.03%)	1.24 (0.81%)	2.67 (0.0%)	202.96 (0.13%)	26.39 (0.02%)
				M3-4-4	33.81%	179	155.36	23.46	23.06 (98.31%)	22.33 (95.19%)	49.93%	153.65	22.83	22.53 (98.67%)	21.84 (95.64%)	49.78%	153.65 (98.9%)	1.23 (0.79%)	5.16 (0.0%)	431.99 (0.28%)	39.9 (0.03%)
**M3-5**	209	57.28	1,114.06	M3-5-1	32.18%	179	145.68	22	21.59 (98.15%)	20.88 (94.9%)	50.31%	144.06	21.43	21.12 (98.53%)	20.44 (95.39%)	50.19%	144.06 (98.89%)	1.35 (0.93%)	6.52 (0.0%)	217.18 (0.15%)	39.54 (0.03%)
				M3-5-2	20.00%	180	127.5	19.25	18.85 (97.92%)	18.15 (94.26%)	49.67%	126.38	18.79	18.46 (98.25%)	17.79 (94.68%)	49.56%	126.38 (99.12%)	953.65 (0.75%)	2.09 (0.0%)	144.22 (0.11%)	20.69 (0.02%)
				M3-5-3	21.31%	184	125.36	18.93	18.48 (97.63%)	17.72 (93.61%)	49.78%	124.17	18.46	18.09 (97.98%)	17.37 (94.06%)	49.66%	124.17 (99.05%)	1.04 (0.83%)	2.14 (0.0%)	136.99 (0.11%)	15.72 (0.01%)
				M3-5-4	39.47%	189	176.12	26.59	26.17 (98.41%)	25.38 (95.43%)	50.55%	174.23	25.97	25.64 (98.73%)	24.89 (95.84%)	50.42%	174.23 (98.93%)	1.38 (0.78%)	5.98 (0.0%)	455.5 (0.26%)	46.78 (0.03%)
**M3-6**	209	57.28	1,114.57	M3-6-1	33.66%	183	176.39	26.64	26.15 (98.19%)	25.3 (94.98%)	50.19%	174.43	25.96	25.59 (98.56%)	24.78 (95.46%)	50.08%	174.43 (98.89%)	1.63 (0.92%)	7.98 (0.0%)	268.95 (0.15%)	54.93 (0.03%)
				M3-6-2	23.04%	178	130.33	19.68	19.22 (97.64%)	18.43 (93.65%)	49.73%	129.08	19.18	18.79 (98.0%)	18.05 (94.12%)	49.61%	129.08 (99.04%)	1.09 (0.83%)	2.24 (0.0%)	142.47 (0.11%)	17.61 (0.01%)
				M3-6-3	21.03%	178	119.83	18.09	17.68 (97.71%)	16.97 (93.81%)	49.67%	118.67	17.61	17.28 (98.07%)	16.61 (94.28%)	49.54%	118.67 (99.03%)	990.14 (0.83%)	2.06 (0.0%)	147.43 (0.12%)	18.37 (0.02%)
				M3-6-4	33.34%	185	172.14	25.99	25.56 (98.35%)	24.77 (95.28%)	50.09%	170.29	25.35	25.01 (98.69%)	24.26 (95.71%)	49.94%	170.29 (98.93%)	1.34 (0.78%)	5.85 (0.0%)	457.09 (0.27%)	45.11 (0.03%)
**M3-7**	209	57.28	1,116.36	M3-7-1	33.59%	176	172.35	26.02	25.6 (98.38%)	24.83 (95.4%)	50.27%	170.85	25.38	25.05 (98.7%)	24.32 (95.81%)	50.17%	170.85 (99.13%)	1.26 (0.73%)	7.58 (0.0%)	193.87 (0.11%)	37.8 (0.02%)
				M3-7-2	23.30%	180	147.04	22.2	21.71 (97.8%)	20.88 (94.04%)	50.11%	145.72	21.63	21.23 (98.15%)	20.44 (94.48%)	50.00%	145.72 (99.11%)	1.15 (0.78%)	2.59 (0.0%)	141.64 (0.1%)	20.78 (0.01%)
				M3-7-3	28.98%	182	191.94	28.98	28.35 (97.83%)	27.27 (94.08%)	50.08%	190.23	28.29	27.77 (98.16%)	26.74 (94.51%)	49.97%	190.23 (99.11%)	1.48 (0.77%)	3.32 (0.0%)	190.29 (0.1%)	26.84 (0.01%)
				M3-7-4	32.29%	172	171.59	25.91	25.5 (98.41%)	24.74 (95.48%)	49.55%	169.84	25.16	24.85 (98.77%)	24.14 (95.94%)	49.37%	169.84 (98.98%)	1.26 (0.74%)	5.81 (0.0%)	432.01 (0.25%)	47.86 (0.03%)
**M3-8**	209	57.28	1,115.56	M3-8-1	33.97%	178	183.39	27.69	27.19 (98.2%)	26.3 (94.97%)	50.18%	181.71	26.98	26.59 (98.55%)	25.75 (95.42%)	50.06%	181.71 (99.08%)	1.41 (0.77%)	8.25 (0.0%)	227.68 (0.12%)	33.71 (0.02%)
				M3-8-2	27.57%	181	145.86	22.02	21.54 (97.79%)	20.71 (94.02%)	50.05%	144.52	21.42	21.03 (98.15%)	20.24 (94.49%)	49.93%	144.52 (99.09%)	1.15 (0.79%)	2.58 (0.0%)	159.39 (0.11%)	22.73 (0.02%)
				M3-8-3	25.82%	179	151.93	22.94	22.44 (97.79%)	21.56 (93.96%)	49.57%	150.59	22.39	21.97 (98.12%)	21.13 (94.39%)	49.45%	150.59 (99.12%)	1.17 (0.77%)	2.62 (0.0%)	148.2 (0.1%)	20.56 (0.01%)
				M3-8-4	35.19%	183	175.31	26.47	26.03 (98.33%)	25.21 (95.25%)	50.19%	173.44	25.83	25.49 (98.68%)	24.72 (95.69%)	50.07%	173.44 (98.93%)	1.46 (0.84%)	5.92 (0.0%)	349.58 (0.2%)	47.83 (0.03%)
**M3-9**	209	57.28	1,115.85	M3-9-1	25.49%	170	137.32	20.74	20.14 (97.15%)	19.27 (92.92%)	50.18%	135.34	20.08	19.61 (97.7%)	18.8 (93.64%)	50.08%	135.34 (98.56%)	1.75 (1.27%)	3.15 (0.0%)	193.05 (0.14%)	35.1 (0.03%)
				M3-9-2	33.22%	191	183.65	27.73	27.09 (97.7%)	26.01 (93.78%)	49.87%	181.88	27.05	26.52 (98.05%)	25.49 (94.24%)	49.76%	181.88 (99.04%)	1.53 (0.83%)	3.17 (0.0%)	212.53 (0.12%)	25.96 (0.01%)
				M3-9-3	28.26%	184	177.55	26.81	26.28 (98.04%)	25.36 (94.61%)	50.36%	176.14	26.17	25.74 (98.35%)	24.87 (95.01%)	50.26%	176.14 (99.2%)	1.22 (0.69%)	3.17 (0.0%)	157.79 (0.09%)	27.68 (0.02%)
				M3-9-4	20.25%	178	136.2	20.57	19.94 (96.95%)	19.04 (92.59%)	49.93%	133.72	19.85	19.37 (97.59%)	18.54 (93.42%)	49.79%	133.72 (98.18%)	2.06 (1.51%)	3.18 (0.0%)	367.97 (0.27%)	51.47 (0.04%)
**M3-10**	209	57.28	1,114.22	M3-10-1	17.06%	178	84.54	12.76	12.27 (96.14%)	11.63 (91.1%)	49.96%	82.55	12.26	11.89 (96.99%)	11.3 (92.22%)	49.86%	82.55 (97.65%)	1.77 (2.1%)	2.16 (0.0%)	176.85 (0.21%)	31.09 (0.04%)
				M3-10-2	28.25%	181	168.73	25.48	24.89 (97.68%)	23.88 (93.75%)	50.08%	167.13	24.81	24.33 (98.05%)	23.38 (94.23%)	49.95%	167.13 (99.05%)	1.4 (0.83%)	2.96 (0.0%)	179.18 (0.11%)	23.05 (0.01%)
				M3-10-3	27.12%	177	147.62	22.29	21.87 (98.12%)	21.13 (94.79%)	49.76%	146.43	21.68	21.34 (98.45%)	20.64 (95.23%)	49.62%	146.43 (99.19%)	995.79 (0.67%)	2.66 (0.0%)	163.2 (0.11%)	29.79 (0.02%)
				M3-10-4	30.76%	186	169.7	25.62	25.18 (98.29%)	24.39 (95.17%)	50.22%	167.88	24.97	24.62 (98.62%)	23.87 (95.6%)	50.08%	167.88 (98.93%)	1.33 (0.79%)	7.53 (0.0%)	429.2 (0.25%)	46.17 (0.03%)
**M3-11**	209	57.28	1,114.80	M3-11-1	24.88%	175	138.48	20.91	20.33 (97.24%)	19.47 (93.1%)	50.10%	136.51	20.28	19.83 (97.77%)	19.02 (93.8%)	50.00%	136.51 (98.57%)	1.73 (1.25%)	3.02 (0.0%)	207.34 (0.15%)	38.64 (0.03%)
				M3-11-2	25.73%	177	142.01	21.44	20.95 (97.69%)	20.11 (93.78%)	49.74%	140.62	20.89	20.48 (98.05%)	19.69 (94.24%)	49.62%	140.62 (99.03%)	1.2 (0.84%)	2.45 (0.0%)	159.07 (0.11%)	21.1 (0.01%)
				M3-11-3	20.59%	178	135.98	20.53	20.08 (97.82%)	19.31 (94.03%)	49.52%	134.84	20.02	19.65 (98.15%)	18.91 (94.46%)	49.41%	134.84 (99.16%)	1.01 (0.75%)	2.37 (0.0%)	102.95 (0.08%)	17.95 (0.01%)
				M3-11-4	29.49%	180	169.38	25.58	25.12 (98.21%)	24.3 (95.0%)	50.10%	167.46	24.89	24.53 (98.57%)	23.76 (95.46%)	49.96%	167.46 (98.87%)	1.43 (0.84%)	7.49 (0.0%)	435.5 (0.26%)	46.23 (0.03%)
**M3-12**	209	57.28	1,115.49	M3-12-1	27.99%	179	143.07	21.6	20.97 (97.07%)	20.05 (92.79%)	50.22%	140.76	20.93	20.44 (97.66%)	19.58 (93.56%)	50.10%	140.76 (98.38%)	2.02 (1.41%)	3.33 (0.0%)	247.33 (0.17%)	45.51 (0.03%)
				M3-12-2	26.61%	186	106.58	16.09	15.79 (98.1%)	15.24 (94.71%)	49.96%	105.72	15.74	15.48 (98.39%)	14.96 (95.09%)	49.86%	105.72 (99.19%)	730.42 (0.69%)	1.9 (0.0%)	109.61 (0.1%)	19.79 (0.02%)
				M3-12-3	31.43%	175	215.68	32.57	31.94 (98.07%)	30.84 (94.71%)	49.98%	213.89	31.78	31.27 (98.38%)	30.23 (95.12%)	49.88%	213.89 (99.17%)	1.51 (0.7%)	3.89 (0.0%)	229.2 (0.11%)	43.28 (0.02%)
				M3-12-4	30.96%	176	174.19	26.3	25.84 (98.23%)	25.0 (95.05%)	50.41%	172.33	25.61	25.24 (98.58%)	24.46 (95.51%)	50.28%	172.33 (98.93%)	1.45 (0.83%)	7.72 (0.0%)	362.48 (0.21%)	44.9 (0.03%)
**M3-13**	209	57.28	1,115.48	M3-13-1	24.46%	181	138.42	20.9	20.27 (96.97%)	19.36 (92.6%)	50.41%	136.16	20.23	19.74 (97.58%)	18.89 (93.4%)	50.30%	136.16 (98.37%)	1.99 (1.44%)	3.25 (0.0%)	223.4 (0.16%)	41.81 (0.03%)
				M3-13-2	24.71%	177	124.47	18.79	18.43 (98.04%)	17.78 (94.59%)	50.02%	123.52	18.38	18.07 (98.34%)	17.45 (94.97%)	49.93%	123.52 (99.24%)	829.4 (0.67%)	2.18 (0.0%)	94.37 (0.08%)	18.48 (0.01%)
				M3-13-3	23.00%	196	184.46	27.85	27.29 (97.97%)	26.29 (94.38%)	49.52%	183.06	27.3	26.82 (98.25%)	25.87 (94.75%)	49.43%	183.06 (99.24%)	1.23 (0.67%)	3.31 (0.0%)	140.47 (0.08%)	24.1 (0.01%)
				M3-13-4	31.62%	182	162.87	24.59	24.17 (98.3%)	23.42 (95.22%)	50.47%	161.17	23.95	23.63 (98.64%)	22.92 (95.67%)	50.35%	161.17 (98.96%)	1.34 (0.82%)	7.39 (0.0%)	302.15 (0.19%)	44.41 (0.03%)
**M3-14**	209	57.28	1,114.13	M3-14-1	34.85%	181	105.79	15.97	15.47 (96.82%)	14.7 (92.04%)	51.27%	104.14	15.47	15.07 (97.39%)	14.36 (92.79%)	51.17%	104.14 (98.44%)	1.47 (1.39%)	2.29 (0.0%)	150.21 (0.14%)	22.85 (0.02%)
				M3-14-2	23.43%	178	103.81	15.68	15.38 (98.09%)	14.85 (94.74%)	50.18%	102.97	15.29	15.05 (98.41%)	14.55 (95.16%)	50.08%	102.97 (99.19%)	718.16 (0.69%)	1.81 (0.0%)	101.41 (0.1%)	19.05 (0.02%)
				M3-14-3	28.95%	180	188.15	28.41	27.74 (97.64%)	26.61 (93.66%)	49.92%	186.3	27.7	27.14 (98.0%)	26.07 (94.12%)	49.81%	186.3 (99.01%)	1.61 (0.86%)	3.28 (0.0%)	215.65 (0.11%)	26.4 (0.01%)
				M3-14-4	31.00%	179	190.54	28.77	28.27 (98.26%)	27.36 (95.09%)	49.97%	188.56	28.04	27.65 (98.59%)	26.79 (95.52%)	49.82%	188.56 (98.96%)	1.49 (0.78%)	8.73 (0.0%)	441.84 (0.23%)	47.52 (0.02%)
**M3-15**	209	57.28	1,114.84	M3-15-1	25.97%	177	141.8	21.41	20.77 (97.03%)	19.85 (92.73%)	50.10%	139.46	20.7	20.21 (97.64%)	19.36 (93.53%)	49.98%	139.46 (98.35%)	2.03 (1.43%)	3.2 (0.0%)	258.58 (0.18%)	43.44 (0.03%)
				M3-15-2	27.41%	189	178.93	27.02	26.5 (98.07%)	25.58 (94.66%)	49.84%	177.46	26.43	26.0 (98.36%)	25.12 (95.04%)	49.74%	177.46 (99.18%)	1.24 (0.69%)	3.27 (0.0%)	185.37 (0.1%)	35.37 (0.02%)
				M3-15-3	22.79%	176	139.73	21.1	20.7 (98.09%)	19.98 (94.7%)	49.55%	138.63	20.61	20.28 (98.39%)	19.6 (95.09%)	49.44%	138.63 (99.21%)	931.46 (0.67%)	2.56 (0.0%)	140.56 (0.1%)	26.05 (0.02%)
				M3-15-4	31.35%	180	156.1	23.57	23.15 (98.23%)	22.4 (95.03%)	49.26%	154.38	22.94	22.62 (98.59%)	21.91 (95.49%)	49.09%	154.38 (98.9%)	1.28 (0.82%)	7.13 (0.0%)	382.15 (0.24%)	44.12 (0.03%)
**M3-16**	209	57.28	1,115.19	M3-16-1	30.96%	179	254.92	38.49	37.09 (96.35%)	34.9 (90.68%)	50.20%	251.4	37.38	36.17 (96.78%)	34.1 (91.25%)	50.11%	251.4 (98.62%)	3.12 (1.22%)	13.01 (0.01%)	335.48 (0.13%)	51.22 (0.02%)
				M3-16-2	28.31%	190	143.88	21.73	21.31 (98.08%)	20.57 (94.68%)	49.88%	142.75	21.26	20.91 (98.37%)	20.21 (95.06%)	49.79%	142.75 (99.21%)	979.63 (0.68%)	2.56 (0.0%)	124.37 (0.09%)	24.71 (0.02%)
				M3-16-3	23.41%	191	83.56	12.62	12.38 (98.08%)	11.94 (94.66%)	50.16%	82.89	12.34	12.14 (98.38%)	11.73 (95.04%)	50.06%	82.89 (99.2%)	573.19 (0.69%)	1.48 (0.0%)	78.23 (0.09%)	14.53 (0.02%)
				M3-16-4	33.89%	186	169.14	25.54	25.09 (98.24%)	24.27 (95.04%)	50.00%	167.33	24.88	24.53 (98.58%)	23.76 (95.48%)	49.85%	167.33 (98.93%)	1.36 (0.8%)	7.66 (0.0%)	400.05 (0.24%)	43.11 (0.03%)
**M3-17**	209	57.28	1,115.01	M3-17-1	28.15%	178	140.07	21.15	20.49 (96.85%)	19.53 (92.35%)	49.99%	137.62	20.46	19.95 (97.48%)	19.07 (93.17%)	49.86%	137.62 (98.25%)	2.14 (1.53%)	3.44 (0.0%)	261.43 (0.19%)	42.38 (0.03%)
				M3-17-2	27.42%	180	163.52	24.69	24.19 (97.98%)	23.32 (94.47%)	49.90%	162.11	24.11	23.7 (98.3%)	22.87 (94.88%)	49.79%	162.11 (99.14%)	1.19 (0.73%)	2.98 (0.0%)	178.7 (0.11%)	29.98 (0.02%)
				M3-17-3	30.55%	181	183.3	27.68	27.16 (98.13%)	26.23 (94.77%)	49.99%	181.84	27.06	26.64 (98.43%)	25.75 (95.16%)	49.89%	181.84 (99.2%)	1.24 (0.68%)	3.28 (0.0%)	183.38 (0.1%)	34.96 (0.02%)
				M3-17-4	18.28%	176	128.67	19.43	18.89 (97.24%)	17.99 (92.62%)	48.94%	126.84	18.85	18.4 (97.65%)	17.56 (93.16%)	48.79%	126.84 (98.58%)	1.42 (1.1%)	6.92 (0.01%)	363.57 (0.28%)	37.75 (0.03%)
**M3-18**	209	57.28	1,114.62	M3-18-1	24.41%	180	139.1	21	20.39 (97.06%)	19.48 (92.76%)	50.12%	136.91	20.33	19.85 (97.64%)	19.02 (93.53%)	50.01%	136.91 (98.43%)	1.92 (1.38%)	3.2 (0.0%)	224.71 (0.16%)	40.85 (0.03%)
				M3-18-2	22.14%	188	100.86	15.23	14.94 (98.13%)	14.43 (94.77%)	49.82%	100.07	14.91	14.67 (98.41%)	14.18 (95.14%)	49.73%	100.07 (99.22%)	677.98 (0.67%)	1.74 (0.0%)	89.47 (0.09%)	18.78 (0.02%)
				M3-18-3	25.70%	181	206.53	31.19	30.27 (97.06%)	28.77 (92.26%)	49.10%	204.34	30.4	29.62 (97.43%)	28.19 (92.74%)	49.00%	204.34 (98.94%)	1.93 (0.94%)	6.88 (0.0%)	207.38 (0.1%)	34.2 (0.02%)
				M3-18-4	23.86%	183	145.04	21.9	21.3 (97.25%)	20.3 (92.67%)	50.06%	143	21.24	20.75 (97.66%)	19.8 (93.21%)	49.95%	143.0 (98.6%)	1.61 (1.11%)	7.71 (0.01%)	373.59 (0.26%)	39.99 (0.03%)

Sequencing quality information of cell lines and mixtures is shown. Total number of reads, bases, number of bases that have quality more than 20 and 30, and GC contents before and after Filtering by fastp. K kilobase,,M megabase, G. gigabase.
